# Bovine Medicine and Surgery

**Published:** 1883-01

**Authors:** 


Bovine Medicine and Surgery: By J. Woodruff Hill, F.R.C.V.S., 650 pages with 153 illustrations. London, Bailliere, Tindall & Cox. 1882. New York, William R. Jenkins, Sixth Avenue. 1882.

This work is in many respects a valuable volume for the library of the veterinary student and practitioner. The profuse quotations from many *haman* and *comparative* pathologists and medical writers renders Professor Hill's book of great benefit to those who do not possess all of the valuable works on comparative medicine and surgery. We have carefully examined the quotations and have not discovered a single instance where proper credit was not given. Prof. Hill manifiested excellent judgment in the extended and appropriate quotations from the best writers. And if he seems to some rather too profuse in his quotations from writings of George Fleming, he could not have paid a more deserving compliment to genius and learning.

The author, in the introductory chapter on "Health and Disease," offers many practical suggestions with reference to the manner of securing and maintaining proper hygenic conditions as necessary factors in the development of the organism. The chapters following are concise, and indicate extensive and intelligent observation on the part of the author. Special attention is given to symptoms and treatment, the pathological condition being demonstrated with unusual clearness, by photolithographs and wood-cuts. Many of the diseases, such as splenic fever and pleuro-pneumonia, are treated of at length, there being no speculative statements made, but simply facts given, such as have been deduced from data furnished by the cases investigated and treated.

On page 153 the author says: "An excellent article on this latter form of stomatitis (gangrenous stomatitis) appears in the VETERINARY JOURNAL January, 1881, from the pen of A. E. Macgillivray, M.R.C.V.S., Banff, N. B., in which he recommends excision of every vestige of the disease, dressing with plu copri sulph, and the administration of sulphate of soda." And again on page 319, he quotes from the same writer on the fatal effects of induration of the cervix uteri of animals, recorded in Fleming's "Veterinary Obstetrics," in which are also mentioned some extremely interesting and successful operations for the same. We mention these because of "Notes on Hill's Book," in the VETERINARY JOURNAL, November, 1882, by A. E. Macgillivray.

The arrangement of Prof. Hill's book is appropriate, the letter-press is plain, the style is easy, flowing, and perspicuous; and the field of *bovine medicine* and *surgery* is thoroughly canvassed. As an entire work it is both creditable to the author and to the publishers, and no library of *comparative medicine* and *surgery* will be complete without it.

One feature of this work, eminently worthy of commendation, is the extensive reference to the most noted anthors of works on various subjects of *human medicine*.

The most expensive part of book-making is in illustrations. Though Prof. Hill's book is not absolutely perfect in any respect, yet in its illustrations, both colored and plain, it is well up to the standard, as compared to works of a similar character of recent date of publication.

Mr. Jenkins has shown excellent judgment in bringing out so valuable a work at this time. New York is fast becoming noted for its schools of Veterinary Medicine, and we especially commend this work to students who desire to increase their knowledge and at the same time enlarge their library by the acquisition of reliable books on the diseases incident to the lower animals.

